# DNA promoter hypermethylation in nipple fluid: a potential tool for early breast cancer detection

**DOI:** 10.18632/oncotarget.8352

**Published:** 2016-03-25

**Authors:** Jolien S. de Groot, Cathy B. Moelans, Sjoerd G. Elias, Mary Jo Fackler, Robert van Domselaar, Karijn P.M. Suijkerbuijk, Arjen J. Witkamp, Saraswati Sukumar, Paul J. van Diest, Elsken van der Wall

**Affiliations:** ^1^ Department of Pathology, University Medical Center Utrecht, Utrecht, The Netherlands; ^2^ Julius Center for Health Sciences and Primary Care, University Medical Center Utrecht, Utrecht, The Netherlands; ^3^ Department of Oncology, Johns Hopkins University School of Medicine, Baltimore, USA; ^4^ Department of Surgery, University Medical Center Utrecht, Utrecht, The Netherlands; ^5^ Department of Medical Oncology, University Medical Center Utrecht, Utrecht, The Netherlands

**Keywords:** nipple fluid, methylation, breast cancer

## Abstract

**Introduction:**

Nipple fluid aspiration provides direct non-invasive sampling of fluid from the mammary ductal system, where the majority of breast cancers originate. DNA promoter hypermethylation (“methylation”) occurs early and at high frequency in breast carcinogenesis, bearing the potential as a biomarker for cancer detection at its earliest stages. We assessed methylation in nipple fluid from breasts of healthy women, of women with sporadic breast cancer and of their contralateral breasts. Our goal was to investigate whether nipple fluid can be used as a reliable methylation biomarker source.

**Methods:**

Methylation levels of 13 genes were analysed by quantitative multiplex-methylation specific PCR (QM-MSP) in nipple fluid samples from breasts of healthy women, and from the affected and contralateral breasts of breast cancer patients.

**Results:**

Methylation analysis of the low-volume nipple fluid samples was feasible. Despite the generally low methylation levels, cancerous and healthy breasts nipple fluid could be discriminated with an area under the receiver operating characteristic curve (AUC) of 0.64 (p<0.01) based on a multivariate model including *AKR1B1, ALX1, RASSF1A* and *TM6SF1*. Within-patient differences between cancerous and contralateral nipple fluid samples were less prominent.

**Conclusions:**

Cancerous nipple fluid contains increased levels of methylation of tumor suppressor genes that potentially could serve as a biomarker for early breast cancer detection.

## INTRODUCTION

Worldwide, breast cancer is the most common cancer in women [1, 2]. Five-year survival rates range from more than 80% in developed countries to less than 40% in developing areas [1]. Early detection improves breast cancer survival, resulting in the implementation of various imaging modalities for screening, mammography being the most commonly applied modality.

An intraductal approach might evolve into an alternative screening method by offering a way to directly sample fluid from the mammary ductal system, where breast cancer originates in the majority of patients. Aspirated fluid contains cells, DNA, RNA and proteins directly derived from the breast ducts and can thereby be a rich source of breast cancer biomarkers [3, 4]. Fluid from the breast can be obtained by invasive techniques like random fine needle aspiration (FNA) or ductal lavage (DL), but nipple fluid can also be obtained in a completely non-invasive way by an oxytocin-assisted nipple fluid aspiration under vacuum (NAF). Besides being less invasive, NAF causes less discomfort and is easier to perform compared to invasive techniques [5]. We have previously shown that, with this technique, nipple fluid can be obtained successfully and without discomfort in healthy women and women at increased risk of breast cancer [6, 7].

To improve breast cancer screening, the detection of DNA promoter hypermethylation (further denoted “methylation”) in nipple fluid could be of added value. Methylation of tumor suppressor genes occurs early and at high frequency in most cancer types [7]. The detection of methylation in ductal fluids is feasible using a very sensitive PCR technique like quantitative multiplex methylation-specific PCR (QM-MSP) [8] and is therefore a promising biomarker for breast cancer screening [4]. Previously, we described that methylation of 11 genes (*RARB, RASSF1, TWIST1, CCND2, ESSR1, SCGB3A1, BRCA1, BRCA2, CDKN2A, APC* and *CDH1*) is involved in carcinogenesis of sporadic and *BRCA1*-associated breast cancer [9]. Subsequently we validated a new set of genes in sporadic breast cancer tissue and demonstrated that the promoters of the *AKR1B1, ALX1, GHSR, GREM1, RASGRF2, SFRP2, TM6SF1* and *TMEFF2* genes were significantly differentially methylated in normal versus malignant breast tissues [10-12]. As a first step to test the screening potential of methylation in nipple fluid, we compared the methylation status of a subset of these genes in nipple fluid samples obtained from breasts of healthy women, and from the affected and contralateral breasts of breast cancer patients.

## RESULTS

### Nipple fluid aspiration and baseline characteristics

**Figure 1:**
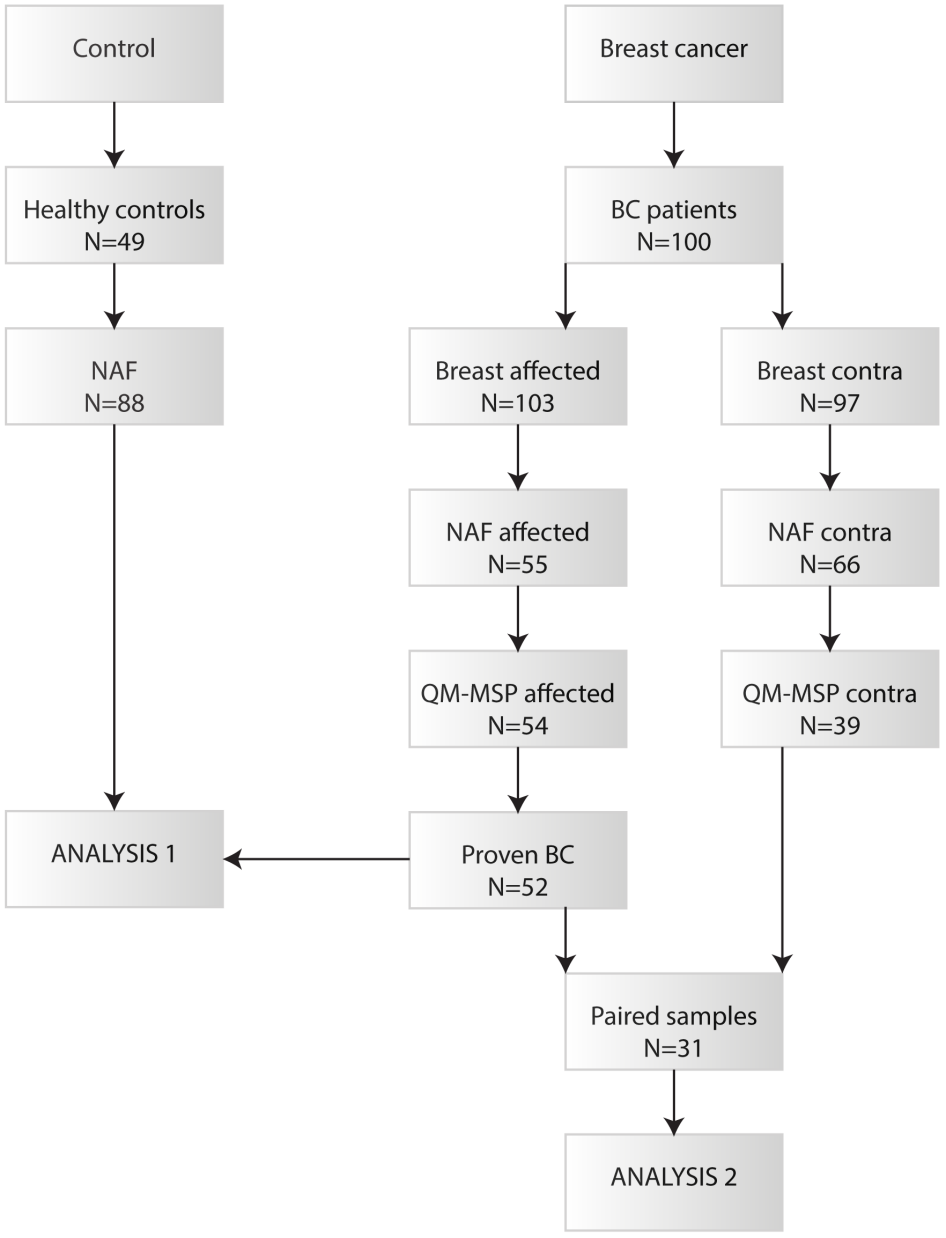
Flow chart of the collected nipple fluid samples that have been analysed by QM-MSP

We used 88 nipple fluid samples from 49 healthy women to set methylation thresholds. In total 100 breast cancer patients were included in the study. In 3 patients the breast cancer was bilateral and one patient was excluded because of missing data on if the yielding breast was affected. Thus, out of 103 attempts to collect nipple fluid from affected breasts and 97 from contralateral breasts, we were able to collect fluid from 55 (53.4%) and 66 (68.0%), respectively. Methylation analysis could be performed in 54 samples from the affected breast and in 39 contralateral samples. After analysis of the surgical specimen, no *in situ* or invasive breast cancer was detected in two women and these samples were consequently excluded from further analysis. Definitive analysis could therefore be performed on 52 breast cancer and 39 contralateral nipple fluid samples, of which 31 were paired. The flow chart of this process is shown in Figure 1.

Baseline characteristics of all included women are shown in Table 1. Mean age of healthy women was 30.7 years (standard deviation (SD) 12.0, median 25.0). In breast cancer patients from whom nipple fluid was collected at the affected or contralateral side, the mean age was 54.6 years (SD 10.4, median 54.0) and 54.9 years (SD 10.0, median 56.0), respectively. The age of affected women significantly differed from the age in the healthy control group (p<0.001 for both). Other clinical parameters correlating with age were also significantly different between these categories, such as history of breast-feeding, parity, oral contraceptive use and menopausal status (p<0.001 for all, in samples with cancer as well as contralateral samples). Moreover, women with breast cancer significantly more often suffered from spontaneous nipple discharge (p=0.024). Pathological characteristics of the breast tumors in the fluid yielding breasts are shown in Supplementary Table S1. In the breast cancer group, one woman carried a *BRCA1* mutation and one woman a *BRCA2* mutation.

**Table 1:** Baseline characteristics of healthy volunteers and breast cancer patients

Feature		Nipple fluid healthy volunteers N=49 (valid %)	Nipple fluid breast cancer N=52 (valid %)	Nipple fluid contralateral breast N=39 (valid %)
Breast size	A-B C-D >D Not available	13 (28.9%) 26 (57.8%) 6 (13.3%) 4	15 (28.8%) 31 (59.6%) 6 (11.5%) 0	11 (28.2%) 21 (53.8%) 7 (17.9%) 0
Spontaneous nipple discharge	No Yes Not available	41 (87.2%) 6 (12.8%) 2	38 (73.1%) 14 (26.9%) 0	28 (71.8%) 11 (28.2%) 0
Breast feeding in history	No Yes Not available	37 (78.7%) 10 (21.3%) 2	23 (44.2%) 29 (55.8%) 0	17 (43.6%) 22 (56.4%) 0
Duration breast feeding	< 6 months 6 – 12 months >12 months Not applicable Not available	2 (4.3%) 7 (14.9%) 1 (2.1%) 37 (78.7%) 2	12 (23.1%) 7 (13.5%) 10 (19.2%) 23 (44.2%) 0	9 (23.1%) 6 (15.4%) 7 (17.9%) 17 (43.6%) 0
Parity	Nulliparous Parous Not available	33 (70.2%) 14 (29.8%) 2	14 (26.9%) 38 (73.1%) 0	7 (17.9%) 32 (82.1%) 0
Age at first birth	<20 years 20 – 24 years 25-29 years >30 years Not applicable Not available	0 (0.0%) 3 (6.4%) 2 (4.3%) 9 (19.1%) 33 (70.2%) 2	4 (7.8%) 8 (15.7%) 10 (19.6%) 16 (31.4%) 13 (25.5%) 1	4 (10.5%) 6 (15.8%) 11 (28.9%) 11 (28.9%) 6 (15.8%) 1
Current use oral contraceptive	No Yes Not available	22 (46.8%) 25 (53.2%) 2	52 (100.0%) 0 (0.0%) 0	39 (100.0%) 0 (0.0%) 0
Current hormonal replacement therapy	No Yes Not available	47 (100.0%) 0 (0.0%) 2	51 (98.1%) 1 (1.9%) 0	39 (100.0%) 0 (0.0%) 0
Age at menarche	≤ 13 years >13 years Not available	25 (54.3%) 21 (45.7%) 3	34 (66.7%) 17 (33.3%) 1	24 (63.2%) 14 (36.8%) 1
Menopausal status	Premenopausal Postmenopausal Not available	41 (87.2%) 6 (12.8%) 2	25 (48.1%) 27 (51.9%) 0	15 (38.5%) 24 (61.5%) 0
Menstrual cycle day	Day 1 – 7 Day 7 – 14 Day 14 – 21 Day 21 – 28 Day >28 IUD Not applicable Not available	13 (27.7%) 5 (10.6%) 14 (29.8%) 5 (10.6%) 4 (8.5%) 0 (0.0%) 6 (12.8%) 2	8 (16.0%) 4 (8.0%) 1 (2.0%) 5 (10.0%) 4 (8.0%) 1 (2.0%) 27 (54.0%) 2	5 (13.2%) 2 (5.3%) 0 (0.0%) 3 (7.9%) 3 (7.9%) 1 (2.6%) 24 (63.2%) 1
Breast cancer in history (invasive or *in situ*)	No Yes Not available	49 (100.0%) 0 (0.0%) 0	44 (84.6%) 8 (15.4%) 0	35 (89.7%) 4 (10.3%) 0

Nipple fluid obtained from the affected or contralateral breasts from breast cancer patients was more often green (23% and 16% versus 2%) or brown/red/bloody (6% and 5% versus 2%) than nipple fluid from healthy women (p<0.001 and p=0.019, respectively). The volume of the aspirated nipple fluid in healthy controls was in 66.0% of the samples up to 10 μl, in 23.4% 10-50 μl, and in 10.6% more than 50 μl. For the affected breasts, these numbers were 72.5%, 23.5% and 4.0%, and for contralateral breasts 59.0%, 30.7% and 10.3%, respectively.

### Methylation in nipple fluid of healthy volunteers versus breast cancer patients

**Figure 2:**
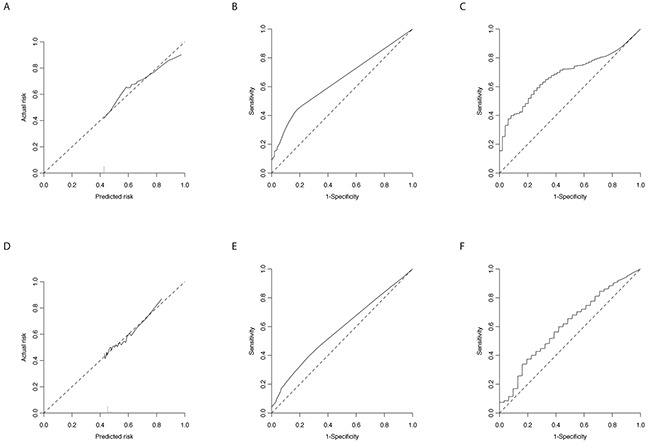
Calibration and ROC curves Calibration **A.** and ROC **B.** curves of the 4 gene model (*AKR1B1*, *ALX1*, *RASSF1A* and *TM6SF1*) and the ROC **C.** curve for the CMI comparing methylation in nipple fluid from healthy controls to cancerous nipple fluid. Graphs **D, E,** and **F.** show the same curves for the analysis of nipple fluid from breasts with breast cancer and the contralateral counterpart.

We used the 90^th^ methylation percentile in the healthy samples as a cut-off for defining the presence of methylation in the nipple fluid samples. This cut-off is shown for each gene in the 13 gene panel in Table 2A, together with the number of missing values, the number of methylated samples in the 52 breast cancer and 49 healthy control samples, the univariable odds ratio (OR) and the corresponding p-value per gene. We included the 4 genes with lowest amount of missing data in the bilateral sample set, i.e. *AKR1B1*, *ALX1*, *RASSF1A* and *TM6SF1*, in multivariable analysis (Table 2B). This panel predicted the presence of breast cancer with an AUC of 0.64 (95%CI 0.54 - 0.74, p<0.01). The graphs of the calibration and ROC curve corresponding to this model are shown in Figure 2A and 2B. At 90% specificity, sensitivity of the 4 gene panel was 31%. Using non-missing (non-imputed) data only, the 4-gene panel was associated with breast cancer occurrence with an AUC of 0.63 (95%CI 0.50 – 0.76, p=0.043). For the CMI, the AUC was 0.68 (95%CI 0.56 – 0.81, p<0.01), see also Figure 2C.

**Table 2:** Methylation analysis comparing nipple fluid of healthy controls and breast cancer patients

A) Univariable analysis for all 13 genes in the original panel							
Gene	Missing (%)	Methylation threshold (%)^a^	Methylation	Breast cancer (N)	Healthy control (N)	Univariable OR (95%CI)	P-value
*MGMT*	14	0.000	Positive Negative	7 45	1 48	6.0 (0.7 – 52.1)	0.1
*RARB*	14	0.583	Positive Negative	12 40	3 46	4.5 (0.9 – 22.3)	0.07
*AKR1B1*	16	0.000	Positive Negative	8 45^b^	2 47	3.3 (0.6 – 16.6)	0.15
*ALX1*	16	1.127	Positive Negative	11 41	5 44	2.4 (0.6 – 8.8)	0.2
*APC*	16	0.021	Positive Negative	6 46	5 44	1.1 (0.2 – 5.3)	0.9
*RASSF1A*	18	0.046	Positive Negative	8 44	3 46	2.7 (0.6 – 12.6)	0.22
*TM6SF1*	21	0.000	Positive Negative	7 45	1 48	7.5 (0.5 – 118.4)	0.15
*CCND2*	26	0.003	Positive Negative	9 43	5 44	2.2 (0.5 – 10.0)	0.32
*SCGB3A1*	26	0.001	Positive Negative	6 46	3 46	1.9 (0.4 – 9.7)	0.43
*GPX7*	34	0.000	Positive Negative	7 45	2 47	3.9 (0.5 – 32.1)	0.21
*NDRG2*	35	0.140	Positive Negative	6 47^b^	4 45	1.2 (0.2 – 7.6)	0.81
*MAL*	39	0.000	Positive Negative	1 51	1 48	1.1 (0.1 – 19.1)	0.92
*GSTP1*	43	0.134	Positive Negative	4 48	3 46	1.1 (0.1 – 11.4)	0.91

a Based on the 90th methylation percentile in healthy nipple fluid samples.

b Numbers after digit were rounded to complete numbers, explaining that the sum of numbers is more than 52.

**Table:** 

B) Multivariable analysis for *AKR1B1*, *ALX1*, *RASSF1A* and *TM6SF1*						
Gene	Methylated breast cancer (%)	Methylated healthy control (%)	Multivariable OR (95%CI)	P-value	AUC (95%CI)	P-value
*AKR1B1*	14	5	2.12 (0.37 – 12.05)	0.39	0.64 (0.54 – 0.74)	<0.01
ALX1	20	10	2.10 (0.54 – 8.22)	0.29		
*RASSF1A*	15	7	2.02 (0.38 – 10.64)	0.41		
*TM6SF1*	13	2	64.34 (0.00 – 3.67 10^14^)	0.78		

### Methylation in nipple fluid of breasts with cancer versus contralateral breasts

Baseline characteristics of the 31 women included in the paired analysis were comparable with the non-selected group of breast cancer patients (data not shown). Mean age of these women was 54.5 years (95%CI 50.4 – 58.5, median 54.0). Table 3A shows the results of univariable analysis for the 13 separate genes in the breast cancer samples versus their contralateral samples. The gene panel consisting of *AKR1B1*, *ALX1*, *RASSF1A* and *TM6SF1* was used in multivariable analysis, of which the results are shown in Table 3B. This panel predicted the presence of breast cancer with an AUC of 0.58 (95%CI 0.44 - 0.72, p=0.26). The corresponding calibration and ROC curves are shown in Figure 2D and 2E. At 90% specificity, sensitivity was 21%. Using non-missing (non-imputed) data only, the 4-gene panel predicted breast cancer occurrence with an AUC of 0.47 (95%CI 0.33 – 0.61, p=0.902). Using the CMI, AUC was 0.61 (95%CI 0.44 – 0.77, p=0.20), see also Figure 2F.

**Table 3:** Methylation analysis comparing nipple fluid of breasts with cancer and their contralateral counterpart

A) Univariable analysis for all 13 genes in the original panel							
Gene	Missing (%)	Methylation threshold (%)^a^	Methylation	Affected side (N)	Contralateral side (N)	Univariable OR (95%CI)	P-value
*AKR1B1*	10	0.000	Positive Negative	4 27	2 29	2.5 (0.3 – 21.3)	0.39
*ALX1*	10	1.127	Positive Negative	5 26	3 28	2.0 (0.3 – 11.6)	0.45
*RASSF1A*	11	0.046	Positive Negative	4 27	3 28	1.5 (0.3 – 8.5)	0.64
*TM6SF1*	16	0.000	Positive Negative	5 26	5 27^b^	1.3 (0.3 – 5.5)	0.75
*CCND2*	18	0.003	Positive Negative	7 24	4 27	2.1 (0.5 – 9.1)	0.35
*SCGB3A1*	18	0.001	Positive Negative	5 26	3 28	2.3 (0.4 – 12.1)	0.34
*RARB*	18	0.583	Positive Negative	6 25	6 25	1.0 (0.2 – 4.4)	1
*APC*	19	0.021	Positive Negative	4 27	5 26	0.8 (0.2 – 4.2)	0.79
*MGMT*	21	0.000	Positive Negative	3 28	3 28	1.2 (0.2 – 9.3)	0.87
*MAL*	37	0.000	Positive Negative	0 31	0 31	1.2 (0.0 – 64.8)	0.92
*NDRG2*	37	0.140	Positive Negative	3 28	5 26	0.5 (0.1 – 3.6)	0.52
*GPX7*	39	0.000	Positive Negative	6 25	1 30	6.5 (0.4 – 95.1)	0.17
*GSTP1*	45	0.134	Positive Negative	2 29	1 30	2.2 (0.1 – 60.8)	0.65

a Based on the 90th methylation percentile in healthy nipple fluid samples.

b Numbers after digit were rounded to complete numbers, explaining that the sum of numbers is more than 31.

**Table:** 

B) Multivariable analysis for *AKR1B1*, *ALX1*, *RASSF1A* and *TM6SF1*						
Gene	Methylated affected side (%)	Methylated contralateral side (%)	Multivariable OR (95%CI)	P-value	AUC (95%CI)	P-value
*AKR1B1*	13	6	2.42 (0.27 – 21.65)	0.43	0.58 (0.44 – 0.72)	0.26
*ALX1*	16	9	1.86 (0.30 – 11.65)	0.51		
*RASSF1A*	14	10	1.43 (0.24 – 8.61)	0.69		
*TM6SF1*	18	15	1.09 (0.23 – 5.13)	0.91		

### Methylation in nipple fluid of breast cancer patients and paired tumor tissue

In general the methylation levels in the tumor tissues were higher than in the nipple fluid samples. To evaluate correlation of methylation values in nipple fluid and corresponding tumor tissue, we calculated the percent of variation in methylation in tumor tissue explained by methylation in nipple fluid. For this analysis, all 52 breast cancer samples were used. Explained variance (R2) was 2.3%, 0.1%, 4.0% and 1.2% for *AKR1B1, ALX1, RASSF1A* and *TM6SF1*, respectively.

## DISCUSSION

By evaluating methylation levels in nipple fluid samples obtained from healthy breasts versus affected and contralateral breasts from patients with breast cancer, this study aimed to investigate whether nipple fluid holds promise as a source of biomarkers for (early) breast cancer detection. We demonstrated that nipple fluid can be obtained from breast cancer patients by aspiration under vacuum, supported by intranasal oxytocin, and that methylation analysis of these low-volume nipple fluid samples is feasible, given that a very sensitive PCR method such as QM-MSP is used [8]. Success rates of obtaining nipple fluid in breast cancer patients were in our experience lower than in healthy women or women at increased breast cancer risk [6, 7]. Since stress decreases lactation performance [13, 14], we expect that stress experienced following breast cancer diagnosis might partly explain the lower success rates in obtaining NAF. Possibly the success rates could be increased by performing more attempts to obtain nipple fluid.

One potential limitation of our study is the age difference between cases and controls possibly leading to methylation differences [15, 16]. In breast cancer, methylation was shown to correlate with age. However, higher age does not necessarily correlate with higher methylation percentages. With increasing age, methylation levels can both in- or decrease [17]. Consequently, only reports on methylation in breast tissue of the genes used in our panel could help to confirm that age-dependency of methylation is a confounder. DNA promoter methylation of a gene panel including *APC*, *CCND2*, *RARB*, *RASSF1A*, and *SCGB3A1* in breast cancer samples was not correlated with age in a previous study [18]. Similar results were shown in normal breast tissue, in which methylation of *RARB*, *RASSF1A*, and *SCGB3A1* hardly correlated to age. In cancer tissue, *SCGB3A1* was only weakly correlated to age, whereas the other genes were not [19]. We previously reported that the CMI of an 11-gene panel (*RARB, RASSF1, TWIST1, CCND2, ESR1, SCGB3A1, BRCA1, BRCA2, CDKN2A, APC, CDH1*) in breast tumors increased with age. However, age-dependency was not determined for the individual genes. When adjusted for age, CMI and the presence of malignancy were still associated [9]. Another complicating factor is that breast cancer risk also increases with age. As a result, it is challenging to discriminate if increasing methylation is due to ageing per se or to the increased breast cancer risk. In normal breast tissue, age-related methylation changes were further altered in breast tumors and may therefore represent early events contributing to breast carcinogenesis [20, 21]. In summary, it is not possible to make definitive assumptions about how age might influence methylation status of our candidate gene panel in nipple fluid or breast tissue. The best way to handle this problem would be to age-match the participants in our nipple fluid studies, but the current sample size does not allow this. Moreover, age is not the only factor possibly influencing methylation status. For example, obesity is associated with methylation in ER-positive tumors [22], making it difficult to ever obtain clinically equal groups of women only differing in breast cancer status.

Even though methylation values in nipple fluid were low, we could discriminate cancerous from healthy nipple fluid samples with an AUC of 0.68 using the CMI, and an AUC of 0.64 using a 4-gene panel. The latter was also predictive without prior data imputation. The differences between cancerous and contralateral nipple fluid samples were less prominent, suggesting that the contralateral breast may undergo field effects and therefore making it more difficult to discriminate between the affected and healthy breast. This is in line with a previous proteomics study that demonstrates a similar protein expression profile in the affected and contralateral breast, but a distinct protein expression profile in healthy breasts [23]. Also for methylation, field effects have been described. In a set of six breast cancer patients, methylation of *RASSF1A* could be found up to 4 cm away from the tumor. Unfortunately, tissue further away was not investigated, but extensive methylation was found in the contralateral breast in two patients [24].

To further validate the critical role of our final 4 gene panel in breast carcinogenesis, we used genomic locations identified in previous studies [10, 11], data generated using the Illumina Human Methylation 27 Beadchip Array, and validated this in The Cancer Genome Atlas (TCGA) Breast Cancer Invasive Carcinoma data (http://cancergenome.nih.gov/). QM-MSP primers were designed to overlap or hybridize to a region within 100 bp of the array probe genomic location. With a methylation threshold of 15%, the frequency of methylation in TCGA breast cancers (N=312) was 64.4% for *AKR1B1*, 67.3% for *ALX1*, 81.4% for *RASSF1A*, and 52.2% for *TM6SF1*. These numbers again stress the importance of methylation of the 4 selected genes/CpG regions in breast carcinogenesis.

Table 4 gives an overview of previous reports describing methylation analysis of nipple aspirate or ductal lavage fluid and demonstrates variable results regarding the diagnostic value of methylation analysis in locally derived breast fluid. Hence, the extent to which the low sample volume and the possible dilution with normal epithelial cells could have contributed to the limited diagnostic accuracy is unclear at this point, and needs to be explored further.

**Table 4:** Literature overview of methylation analysis in ductal lavage/nipple fluid

Author, year	Source of fluid	No. of cases	No. of controls	Used assay	Genes tested	Results
Krassenstein, 2004 [43]	Nipple	22 BC patients	5 healthy and 5 benign disease	MSP	*GSTP1, RAB2, CDKN2A* (p16INK4a, p14ARF), *RASSF1A, DAPK*	Once the individual tumor is positive for hypermethylation of a candidate gene, corresponding fluid was analysed for hypermethylation of that particular gene. Hypermethylation of one or more genes was found in all tumor DNAs and identical gene hypermethylation was detected in 82% matched nipple fluid. Hypermethylation was absent in healthy women.
Fackler, 2006 [8]	Ductal	27 BC patients	3	QM-MSP	*RASSF1A, TWIST, SCGB3A1, CCND2, RARB, APC1, BRCA1, BRCA2, CDKN2A* (p16INK4a)	Sensitivity of 62% and specificity of 82% for detecting breast cancer by CMI.
Euhus, 2007 [44]	Ductal	67 BC patients	83	QM-MSP	*CCND2, APC, SCGB3A1, RASSF1A, RARB2*	QM-MSP data available for 34 tumor-tissue FNAs from patients participating in the ductal lavage study. Frequency of methylation was significantly higher in tumors than corresponding fluid for every gene. Among methylation-positive cases, median methylation fraction was significantly increased for tumor tissue than corresponding fluid for *APC, HIN1*, and *RASSF1A*. There was no correlation between individual methylation fractions of tumors as compared to corresponding fluid for any gene.
Locke, 2007 [45]	Ductal	7 *BRCA1*, 12 *BRCA2* mutation carriers	5	MSP	*RARB, SCGB3A1, TWIST, CCND2*	In 42% of *BRCA* mutation carriers, at least one gene was hypermethylated, but no hypermethylation was observed control patients (p=0.13).
Antill, 2010 [46]	Ductal	16 *BRCA1*, 18 *BRCA2* mutation carriers;7 developing BC (1 bilateral)	None	QM-MSP	*CDKN2A (p16INK4a), RASSF1A, TWIST, RARB*	Methylation was detected in 61% of *BRCA* mutation carriers. Methylation of *CDKN2A* (p16INK4a) was observed in 37% that coincided with a BRCA1 mutation, *RASSF1A* methylation in 24%, *TWIST* methylation in 6% that was associated with cytologic atypia, and *RARB* methylation in 24% that coincided with an association between NAF production and methylation in this gene at individual breast level.
Zhu, 2010 [47]	Nipple; Ductal	18; 61 BC and 10 precancer	None; 48 benign disease	Q-MSP	*CCND2, CDKN2A (p16INK4a), RARB, RASSF1A*	NAF versus normal tissue showed only a significant difference in hypermethylation of *CDKN2A* (p16INK4a); significant difference in cancer compared to benign ductal lavage for p16 and *RASSF1*. No significant difference between cancer and precancer samples as well as between precancer and benign samples.
Twelves, 2013 [48]	Ductal	54	46 contralateral	MSP	*RASSF1A, SCGB3A1, PDLIM4, CDH13, RARB, IGFBP7*	Significant difference in hypermethylation of *SCGB3A1, CDH13, RARB* and *IGFBP7* between breast cancer and healthy samples. ROC 0.76, false positive 33%, false negative 16%.

BC= breast cancer; NAF= nipple aspiratin fluid

Apart from breast fluids, methylation as a biomarker has been studied in different other types of body fluids, such as vaginal swabs [25], urine [26], or stool samples [27], generally yielding much better results. Moreover, studies with other, high-volume biofluids show a better correlation between fluid and tumor tissue, e.g. in urine [28] and stool [29]. The larger volume of the samples in these studies compared to nipple fluid may have contributed to the higher reported diagnostic accuracies. Moreover, vaginal swabs and urine provide a more direct way of sampling from the source organ, whereas usually six to eight ducts reach the human nipple and it remains elusive whether shedded epithelial cells and DNA from the cancerous duct are efficiently collected in the obtained nipple fluid. Although there is evidence for a methylation field defect in breast cancer, nipple fluid samples may be diluted with non-diseased material. This could account for the low correlation observed between methylation in nipple fluid and the corresponding breast cancer tissue. Collecting fluid via ductal lavage might improve diagnostic accuracy by providing a larger sample volume and allowing more direct sampling of the affected ducts, at the costs of a more invasive procedure being less applicable in a screening setting. In addition, other classes of biomarkers may improve diagnostic accuracy such as proteins [30-32], hormones [33-35], lipids [36] or microRNAs.

In conclusion, we have clearly demonstrated that cancerous nipple fluid contains increased levels of methylation biomarkers that may help to detect breast cancer in a non-invasive way. As part of a large prospective trial in cooperation with the Erasmus Medical Center, the Netherlands, we are currently yearly collecting nipple fluid of women at increased breast cancer risk for 10 years, resulting in a valuable biobank of nipple fluid samples. In the future, this biobank will allow us to test the predictive value of nipple fluid biomarkers in a prospective setting, so appropriate preventive measures can be taken.

## MATERIALS AND METHODS

### Nipple fluid and tissue samples

The clinical study collecting nipple fluid from healthy women by aspiration under vacuum, supported by intranasal oxytocin, was described previously [6]. Similarly, we collected nipple fluid from sporadic breast cancer patients using a cross-sectional study design between 2010 and 2014. Women were included when having proven or suspected breast cancer based on biopsy, either invasive or *in situ*. Exclusion criteria were age under 18 years, bilateral ablative breast surgery, pregnancy or lactation, having an active breast infection and/or having disseminated breast cancer. Nipple fluid was collected prior to breast cancer surgery from the affected and contralateral breast as described before [6]. TNE buffer (50 mM Tris pH 8.0, 150 mM NaCl, 2 mM EDTA) was added and the nipple fluid sample was stored at −80°C until further analysis blinded for breast cancer status. Formalin-fixed paraffin-embedded (FFPE) breast cancer tissue, collected at the time of surgery, was used after diagnostics had been completed. The study was approved by the Internal Review Board of the UMC Utrecht, the Netherlands (ABR NL 11690.041.06, METC 06-091). Written informed consent was obtained from all participants.

### DNA extraction

DNA was isolated from nipple fluid samples using the High Pure Viral Nucleic Acid kit (Roche, 11858874001) and low-retention Eppendorf tips (Biotix, 2012091). Preferably 10 μl of the sample was used for DNA isolation, and DNA was isolated twice for duplicate analysis. If less than the required amount was available, all nipple fluid was used.

For DNA isolation from FFPE tissue, one to five 10 μm unstained sections were deparaffinized in xylene and rehydrated through a series of alcohol. Relevant tissue, as indicated by a pathologist on sandwich H&E stained sections, was scraped from the slide and 100 μl lysis buffer (0.5% Tween-20, 50 mM Tris pH 8.5) containing 20-40 μg proteinase K (Invitrogen) was added. After incubation at 56°C overnight, the reaction was heat inactivated for 10 min at 95°C and centrifuged at 14,000 rpm for 3 min. The supernatant was transferred to a new tube and DNA concentration as well as 260/280 absorbance were measured with a spectrophotometer (NanoDrop ND-1000, Thermo Scientific). Samples were stored at 4°C until further analysis.

### Sodium bisulfite conversion

Sodium bisulfite conversion was performed using the Epitect bisulfite kit (Qiagen, 59104) according to the protocol “Sodium Bisulfite Conversion of Unmethylated Cytosines in Small Amounts of Fragmented DNA” (protocol version 09/2009) with an input of 40 μl of DNA from nipple fluid and 1.5 μg DNA from tissue. Human sperm DNA was used as a negative control and *Sss*I methylase treated MDA-MB-231 gDNA as a positive control. A non-template control and 3% methylated control were included in each bisulfite conversion reaction.

### External multiplex PCR

Immediately after sodium bisulfite treatment, multiplex PCR was performed with 12 μl of converted DNA kept on ice and a primer mix to amplify the following 13 genes: *AKR1B1, ALX1, APC1, CCND2, GPX7, GSTP1, HIN1, MAL, MGMT, NDRG2, RARB, RASSF1A*, and *TM6SF1*. This set of genes was chosen after validation in breast cancer tissue [9-12] and based on their initial performance in nipple fluid samples. Primer sequences are shown in Supplementary Table S2. For each PCR reaction, converted DNA was added to 13 μl of reaction mix consisting of 1x MSP buffer (67 mM Tris (pH 8.8), 6.7 mM MgCl_2_, 10 mM β-mercaptoethanol, 0.1% DMSO), 1.25 mM dNTPs, 2.5 units of Platinum Taq (Invitrogen) and 0.1 μM each of the forward and reverse primers (dissolved in distilled water containing 50 μg/ml Salmon Sperm DNA). PCR conditions were 95°C for 5 minutes, followed by 40 cycles of 95°C for 30 seconds, 56°C for 45 seconds and 72°C for 45 seconds, with a final extension cycle of 72°C for 7 minutes. PCR products were diluted, 500x for NAF and 500-5000x for tissue, in dilution buffer (distilled water containing 1x MSP buffer and 100 μmg/ml Salmon Sperm DNA) for further analysis.

### Gene-specific quantitative PCR (Q-PCR)

To investigate the specific target gene of interest, a nested real-time methylation specific PCR (QM-MSP) [8] was performed for each candidate gene separately. Used primer and probe sequences are listed in Supplementary Tables S3 and S4. For each reaction, 4 μl of diluted multiplex PCR product was added to 16 μl μf reaction mix consisting of 1x MSP buffer, 200 μM dNTPs, 500 nM ROX 50 (Invitrogen), 1 unit of Platinum Taq (Invitrogen), 400 nM of each primer, and 400 nM of each probe. Q-PCR was performed on the Applied Biosytems ViiA7 Real-Time PCR System with the following conditions: 50°C for 2 minutes, 95°C for 7 minutes, followed by 50 cycles of 95°C for 15 seconds and 65°C for 1 minute. For each gene, a standard dilution curve (dilution 10^−2^, 10^−3^, 10^−4^, 10^−5^, 10^−6^, 10^−7^) of a mixed sample containing fully methylated *Sss*I methylase treated MDA-MB-231 gDNA and unmethylated human sperm DNA in a 1:1 ratio was included for extrapolating percent methylation. Analysis was performed blinded for tumor status.

### Statistical analysis

As methylation status could not be assessed for all genes in all samples, and since disregarding missing data may lead to biased results and loss of information [37, 38], we imputed missing data by the multivariate imputation by chained equations (MICE) method (20 datasets) [39]. We used extensive information for imputing missing data: baseline characteristics including reproductive factors, cancer status of the breast, tumor characteristics if applicable and methylation percentages (Table 1, Supplementary Table S1). All analyses were performed in each imputed dataset separately and pooled using Rubin's rules.

After imputation, we first compared methylation between healthy volunteers and patients with breast cancer. For this, we used nipple fluid samples collected unilaterally in healthy volunteers (N=49) and nipple fluid from breast cancer patients (N=52; affected side). In case of two samples from the same woman in the control group, we randomly selected one of the samples. Second, methylation between the affected and paired contralateral breasts was compared (N=31). Finally, methylation in nipple fluid was compared to methylation status in tumor tissue of the same breast (N=52).

To compare methylation values of the nipple fluid samples, methylation signals were evaluated as a dichotomous variable, based on the 90^th^ percentile in the healthy control group (N=88). The threshold of the 90^th^ percentile was based on a study analysing methylation in ductal fluid [8]. To begin with, we evaluated the discriminating value of each gene in a univariable comparison. For multivariable logistic regression analysis, we selected 4 genes, since less than 10 events per variable statistical models may not be valid [40, 41]. We chose the 4 genes with the lowest amount of missing data in the bilateral sample set (N=31). We used the same panel of genes in the comparison of healthy versus breast cancer samples, in order to make all analyses comparable although this analysis formally allowed for more genes to be evaluated.

Next, we evaluated the predictive performance of each logistic regression model for discrimination and calibration. Discrimination was assessed with the area under the receiver operating characteristic (ROC) curve (AUC). To evaluate how close the model-derived predicted cancer probabilities reflected observed probabilities over the entire range of possible values, we made use of calibration plots. To validate the results of the 4-gene panel in non-imputed data, we performed multivariable logistic regression and ROC analysis of the cases with non-missing methylation values for *ALX1, AKR1B1, RASSF1*, and *TM6SF1* genes. The number of complete cases was 29, 40 and 28 for healthy, cancerous and contralateral nipple fluid samples, respectively.

In addition to using dichotomous variables, we also calculated the sum of the methylation percentages of all 13 analysed genes, the cumulative methylation index (CMI) [42]. Also for CMI, an AUC was calculated.

To evaluate correlation of methylation values in nipple fluid and corresponding tumor tissue, we calculated the percent of variation in methylation in tumor tissue explained by methylation in nipple fluid.

Statistical analyses were performed using IBM SPSS Statistics version 20.0 (SPSS, Inc., Chicago, Il, USA) and R version 3.1.1 (The R foundation for Statistical Computing, Vienna, Austria, http://www.R-project.org/) including package Regression Modeling Strategies (http://CRAN.R-project.org/package=rms). We considered a two-sided p-value <0.05 as statistically significant, and report parameter estimates with 95% confidence intervals (CI).

## SUPPLEMENTARY TABLES


