# Supplementary material for: DNA promoter hypermethylation in nipple fluid: a potential tool for early breast cancer detection
Source: Oncotarget. 2016 Mar 25;7(17):24778–91. doi: 10.18632/oncotarget.8352 (PMC5029741; doi:10.18632/oncotarget.8352)
Supplement: Supplementary file 1 [file oncotarget-07-24778-s001.pdf]

SUPPLEMENTARY TABLES

Supplementary Table S1: Clinicopathological characteristics of breast tumors of which corresponding nipple fluid was collected (N=52)

Feature		Number of tumors (%)
Invasiveness	Ductal carcinoma *in situ*	6 (11.5%)
	Invasive carcinoma	46 (88.5%)
Histologic type	Ductal	32 (69.6%)
	Lobular	4 (8.7%)
	Ductolobular	7 (15.2%)
	Tubular	2 (4.3%)
	Mucinous	1 (2.2%)
Histologic grade	1	9 (19.6%)
	2	20 (43.5%)
	3	12 (26.1%)
	Unknown	5 (10.9%)
ERα	Negative	6 (13.0%)
	Positive	39 (84.8%)
	Unknown	1 (2.2%)
PR	Negative	13 (28.3%)
	Positive	32 (69.6%)
	Unknown	1 (2.2%)
Her2	Negative	35 (76.1%)
	Positive	10 (21.7%)
	Unknown	1 (2.2%)
Lymph node	Negative	24 (52.2%)
	Positive[a]	21 (45.7%)
	Unknown	1 (2.2%)

[a] Including isolated tumor cells (4) and micro-metastases (6).

Supplementary Table S2: Primer sequences of multiplex PCR

Gene	Forward primer sequences 5’ to 3’	Reverse primer sequences 5’ to 3’
AKR1B1	GYGTAATTAATTAGAAGGTTTTTT	AACACCTACCTTCCAAATAC
ALX1	AGTAAAGYGTTTGTAGGTAAAT	TTTCTCCCTCCCTACCTAAT
APC1	AAAACCCTATACCCCACTAC	GGTTGTATTAATATAGTTATATGT
CCND2	TATTTTTTGTAAAGATAGTTTTGAT	TACAACTTTCTAAAAAATAACCC
GPX7	GGTGAAATTGAGGTTTAGAG	ATACTTCTCCAACRACACCA
GSTP1	TTGGGAAAGAGGGAAAGG	TAACCCCATACTAAAAACTCTAAAC
HIN1	GTTTGTTAAGAGGAAGTTTT	AAACTACAAAACAAAACCAC
MAL	GATTTATAGTTTTTAGTTTTGGA	AAACCACTAAACAAAATACTAC
MGMT	GTTTYGGATATGTTGGGATAGTT	ATAAAAACRCCTACAAAACCACTC
NDRG2	GGGAYGTAGTAAAGAGAGG	CCCAAACTCAATAATAAAAACC
RARB	GTAGGAGGGTTTATTTTTTGTT	AATTACATTTTCCAAACTTACTC
RASSF1A	GTTTTATAGTTTTTGTATTTAGG	AACTCAATAAACTCAAACTCCC
TM6SF1	AGGAGATATYGTTGAGGGGA	TCACTCATACTAAACCRCCAA

Supplementary Table S3: Quantitative PCR primer sequences

Gene	M/U[a]	Forward primer sequences 5' to 3'	Reverse primer sequences 5' to 3'
AKR1B1	M	GCGCGTTAATCGTAGGCGTTT	CCCAATACGATACGACCTTAAC
	U	TGGTGTGTTAATTGTAGGTGTTTT	CCCAATACAATACAACCTTAACC
ALX1	M	CGTGCGTTTGGAGAGGATTTC	CGATCCTACATTTTCGATTACGA
	U	ATGTGTGTTTGGAGAGGATTTTG	ACCAATCCTACATTTTCAATTACAA
APC	M	AATACGAACCAAAACGCTCCC	TATGTCGGTTACGTGCGTTTATAT
	U	TAAATACAAACCAAAACACTCCC	GTTATATGTTGGTTATGTGTGTTT
CCND2	M	TTTGATTTAAGGATGCGTTAGAGTACG	ACTTTCTCCCTAAAAACCGACTACG
	U	TTAAGGATGTGTTAGAGTATGTG	AAACTTTCTCCCTAAAAACCAACTACAAT
GPX7	M	ACGGTGGTAGCGGCGTGGTT	ACCCCGAATATTAACCGCCTTA
	U	TGATGGTGGTAGTGGTGTGG	ACCCCAAATATTAACCACCTTAA
GSTP1	M	TCGGCGTCGTGATTTAGTATTG	AACTACGACGACGAAACTCCAA
	U	GTtGGtGTtGTGATTTAGTATTG	AAACTACaACaACaAAACTCCAAC
HIN1	M	TAGGGAAGGGGGTACGGGTTT	CGCTCACGACCGTACCCTAA
	U	AAGTTTTTGAGGTTTGGGTAGGGA	ACCAACCTCACCCACACTCCTA
MAL	M	TTTCGCGGAGTTAGCGAGAG	AAACCATAACGACGTACTAACG
	U	GTTTTGTGGAGTTAGTGAGAGG	AAACCATAACAACATACTAACATC
MGMT	M	TTTCGACGTTCGTAGGTTTTCGC	GCACTCTTCCGAAAACGAAACG
	U	TTTGTGTTTTGATGTTTGTAGGTTTTTGT	AACTCCACACTCTTCCAAAAACAAAACA
NDRG2	M	AGAGGTATTAGGATTTTGGGTACG	GCTAAAAAAACGAAAATCTCGC
	U	AGAGGTATTAGGATTTTGGGTATGA	CCACTAAAAAAACAAAAATCTCACC
RARB	M	AGAACGCGAGCGATTCGAGTAG	TACAAAAAACCTTCCGAATACGTT
	U	TTGAGAATGTGAGTGATTTGAGTAG	TTACAAAAAACCTTCCAAATACATTC
RASSF1A	M	GCGTTGAAGTCGGGGTTC	CCCGTACTTCGCTAACTTTAAACG
	U	GGTGTTGAAGTTGGGGTTTG	CCCATACTTCACTAACTTTAAAC
TM6SF1	M	CGTTTAGCGGGATGCGGTGA	ACACGAAAACCCCGATAACCG
	U	TGTTTAGTGGGATGTGGTGAAG	ACACAAAAACCCCAATAACCACA

[a] M, methylation-specific primer sequences; U, unmethylated-specific primer sequences.

Supplementary Table S4: Quantitative PCR probe sequences

Gene	Methylation-specific probe sequences 5’ to 3’	Unmethylated-specific probe sequences 5’ to 3’
AKR1B1	HEX-CGTACCTTTAAATAACCCGTAAA ATCGA -BHQ1	6FAM-ACATACCTTTAAATAACCCATA AAATCAAC-BHQ1
ALX1	HEX-AACGCTAACGACTCACCGCTACTAT -BHQ1	6FAM-TAAAACACTAACAACTCAC CACTACTAT-BHQ1
APC1	HEX-CCCGTCGAAAACCCGCCGATTA -BHQ1	6FAM-TTCCCATCAAAAACCCACC AATTAAC-BHQ1
CCND2	HEX-TCGCCGCCAACACGATCGACCCTA -BHQ1	6FAM-TCACCACCAACACAATCAA CCCTAAC-BHQ1
GPX7	HEX-TACTACGCGCAAACCGCAACCCAC -BHQ1	6FAM-CTACTACACACAAACCACA ACCCAC-BHQ1
GSTP1	VIC/HEX-CGAACTCCCGCCGACCCCAACC-TAMRA/BHQ1[A]	6FAM-CAAACTCCCACCAACCCC AACCCC-TAMRA/BHQ1[A]
HIN1	HEX-ACTTCCTACTACGACCGACGAACC -BHQ1	6FAM-CAACTTCCTACTACAACCA ACAAACC-BHQ1
MAL	HEX-AAAACGAAACGAACGCCGCTCAAAC -BHQ1	6FAM-CTTAAAACAAAACAAACACCA CTCAAAC-BHQ1
MGMT	HEX-CACCAAATCGCAAACGATACGCACC -BHQ1	6FAM-CTCACCAAATCACAAACAATAC ACACC-BHQ1
NDRG2	HEX-AAACGCAAAAATTCCGACTCCCTC -BHQ1	6FAM-CACAAAAATTCCAACTCC CTCATAC-BHQ1
RARB	HEX-ATCCTACCCCGACGATACCCAAAC -BHQ1	6FAM-AAATCCTACCCCAACAATAC CCAAAC-BHQ1
RASSF1A	HEX-ACAAACGCGAACCGAACGAAACCA -BHQ1	6FAM-CTAACAAACACAAACCAAACAA AACCA-BHQ1
TM6SF1	HEX-AAACACTCATCGCAACCGCCGCG -BHQ1	6FAM-AAACACTCATCACAACCAC CACACC-BHQ1

[a] All nipple fluid samples were analyzed using FAM-TAMRA and VIC-TAMRA, tumor tissue samples with FAM-BHQ and HEX-BHQ. Comparing studies did not reveal different results.
